# Risk factors for radiation pneumonitis following adjuvant radiotherapy for breast cancer: a retrospective cohort study

**DOI:** 10.3389/fonc.2026.1882033

**Published:** 2026-07-13

**Authors:** Chuou Yin, Jiang Liu, Juan Deng, Guojian Mei, Yingying He, Hui Liu, Hao Cheng

**Affiliations:** Department of Oncology, Deyang People’s Hospital, Deyang, China

**Keywords:** breast cancer, multivariate analysis, radiation pneumonitis, radiation therapy, univariable analyses

## Abstract

**Objective:**

To delineate the risk factors associated with radiation pneumonitis (RP) in breast cancer patients receiving adjuvant radiotherapy, thereby informing strategies for dosimetric optimization and risk mitigation.

**Methods:**

A retrospective analysis was performed on 811 female patients who underwent postoperative radiotherapy for breast cancer to investigate the potential risk factors associated with RP. Specifically, univariate analysis was employed to identify factors associated with RP, followed by multivariate logistic regression modeling to determine independent risk factors. Receiver operating characteristic (ROC) curve analysis was performed to evaluate the predictive performance of identified risk factors, with diagnostic accuracy quantified by the area under the curve (AUC).

**Results:**

Univariate analysis demonstrated that the tumor stage, chest wall + supraclavicular + internal mammary lymph node irradiation (CSI), the number of chemotherapy cycles, the ipsilateral lung V_5_, V_10_, V_20_, and mean lung dose (MLD) were all significantly associated with RP (all *P* < 0.05). Multivariate analysis identified MLD (odds ratio [OR] = 11.136, 95% confidence interval [CI]: 5.494 ∼22.571; *P* < 0.001), the number of chemotherapy cycles (OR = 2.739, 95% CI: 1.597 ∼ 4.696; *P* < 0.001) and CSI (OR =5.654, 95% CI: 1.768 ∼ 18.080; *P* = 0.003) as risk factors for RP. With a diagnostic threshold of MLD = 12.70 Gy, the predictive sensitivity and specificity for RP were 0.898 and 0.965, respectively. Setting the number of chemotherapy cycles at 7 as the threshold yielded a sensitivity of 0.898 and specificity of 0.587 for RP prediction. For CSI, the predictive sensitivity and specificity were 0.878 and 0.810, respectively.

**Conclusions:**

The MLD, the number of chemotherapy cycles and CSI were confirmed as risk factors for RP. Bootstrap internal validation confirmed the robustness of these findings. Stringent dosimetric constraints to minimize MLD are imperative, particularly in patients with extended chemotherapy histories (≥ 7 cycles) undergoing CSI.

## 1 Introduction

Breast cancer is one of the leading threats to women’s health globally, an estimated 670,000 deaths worldwide were attributed to breast cancer in 2022 (1, 2). Multimodal therapy for breast cancer typically encompasses surgical resection, radiotherapy, chemotherapy, and endocrine therapy. Radiation therapy plays a pivotal role in breast cancer management, with approximately 70% of breast cancer patients receiving radiotherapy as part of their treatment regimen (3–5).

Despite therapeutic advances, incidental irradiation of adjacent normal tissues remains inevitable, predisposing patients to radiation-induced injury. Radiation pneumonitis (RP) typically manifests within 1 to 6 months post-treatment completion, representing an unavoidable complication in thoracic tumor radiotherapy (6, 7). The clinical grading criteria for RP are fundamentally anchored in the impact on patients’ pulmonary function, which demonstrates a positive correlation with the volume of lung tissue injury (8). The Common Terminology Criteria for Adverse Events (CTCAE) 5.0 developed by the National Cancer Institute (NCI) classifies the severity of RP as follows: Grade 1 (mild) is characterized by asymptomatic or mild symptoms that are detected exclusively through clinical examination or imaging, and no treatment intervention is required.​ Grade 2 (moderate) refers to moderate symptoms that necessitate local or non-invasive treatment such as oxygen therapy.​ Grade 3 (severe) is defined by severe symptoms and may require oxygen therapy or bronchodilator administration.​ Grade 4 (life-threatening) denotes a critical condition that poses an immediate threat to life, requiring urgent interventions such as mechanical ventilation or endotracheal intubation.​ Grade 5 (death) is classified when the demise of the patient is directly attributable to the adverse event (9).

Investigation of RP specifically in the breast cancer population has been limited by its relatively low incidence, predominantly mild (Grade 1–2) presentation, and challenges in long-term follow-up. However, with prolonged survival outcomes in breast cancer, mitigation of treatment-related toxicity is paramount for preserving long-term quality of life. Consequently, while ensuring radiotherapy plans meet clinical endpoints, dosimetrically targeted optimization thereof is indispensable for mitigating both the incidence and severity of RP.

Prior studies have demonstrated that potential relevant factors for RP include the number of chemotherapy cycles, chemotherapy regimen, history of lung disease, age, chest wall + supraclavicular + internal mammary lymph node irradiation (CSI), surgical approach, chemo-radiotherapy interval, tumor stage, and dose parameters of the ipsilateral lung (10–16). A case report published in the *New England Journal of Medicine* highlighted that V_20_ and mean lung dose (MLD) of the ipsilateral lung may serve as key metrics for RP, and advocated for the assessment of cumulative effects in low-dose regions (V_5_ and V_10_) in clinical practice (10). A multicenter study published in *JAMA Oncology* demonstrated that MLD and V_20_ of the ipsilateral lung are associated with acute skin toxicity, thereby indirectly supporting their potential role as predictors of pulmonary toxicity (12). The IMPORT HIGH trial, published in *Lancet Oncology*, demonstrated that restricting lung high-dose regions (V_20_ < 25%) reduces the risk of RP. In subgroup analysis, patients with (V_5_ ≥ 45.9%) and (V_10_ ≥ 29.4%) had a significantly higher incidence of RP (HR = 1.8), 95% CI: 1.1 ~ 3.0), indicating that low-dose region parameters should be integrated into assessment (13).

This study aims to comprehensively identify factors associated with RP in a cohort of breast cancer patients receiving postoperative radiotherapy. We performed univariate analysis on parameters including chemotherapy cycle number, CSI, chemotherapy regimens, age, surgical method, chemotherapy-radiotherapy interval time, tumor stage, V_5_, V_10_, V_20_ and MLD of the ipsilateral lung, and the volume of planning target volume (PTV) to identify factors associated with RP in breast cancer patients receiving postoperative radiotherapy. Furthermore, multivariate logistic regression analysis will be performed to identify independent risk factors for RP and evaluate the predictive value of each factor.

## 2 Materials and methods

### 2.1 Patients selection

A total of 1,027 female breast cancer patients who received radiotherapy at our department between July 2021 and December 2024 were retrospectively enrolled. Following rigorous screening, patients were excluded if they met any of the following criteria: PTV involving breast prostheses; bilateral breast cancer; concurrent severe cardiovascular or pulmonary diseases (e.g., congenital heart disease, coronary artery disease, cardiomyopathy, chronic obstructive pulmonary disease, asthma, pulmonary tuberculosis, pulmonary fibrosis, etc.); follow-up period < 6 months or loss to follow-up; history of smoking; concurrent autoimmune diseases (e.g., rheumatoid arthritis, systemic lupus erythematosus, etc.) or acquired immunodeficiency syndrome (AIDS).

**Figure 1:**
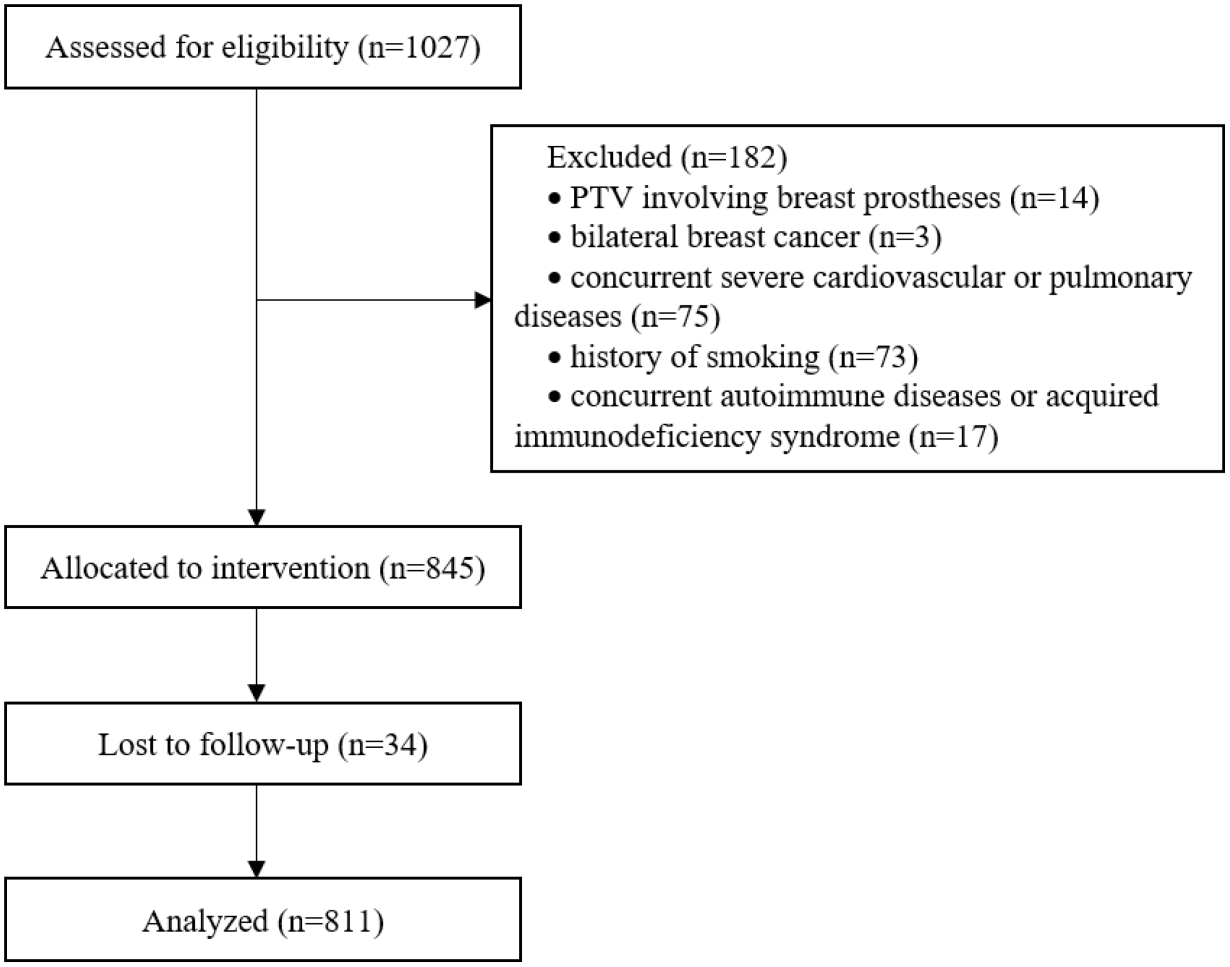
The CONSORT flow diagram of this study.

The CONSORT flow diagram of this study presented in **Figure 1**.

A total of 811 patients were ultimately enrolled in this study. The baseline characteristics of patients with breast cancer in this study were summarized in **Table 1**.

**Table 1:** Baseline characteristics of patients with breast cancer.

Variables	Numbers (%)
Age (27 ∼ 75 years old, with a median age of 52 years), years	
≤52	429 (52.90%)
>52	382 (47.10%)
Gender	
Female	811 (100.00%)
Male	0 (0.00%)
Tumor stage^1^	
I	212 (26.14%)
II	348 (42.91%)
III	251 (30.95%)
Chemotherapy cycles (3∼9 cycles, with a median of 6 cycles), cycles	
≤6	452 (55.73%)
>6	359 (44.27%)
Chemotherapy-radiotherapy interval time (21∼28 days), days	
21 ∼ 22	138 (17.02%)
23 ∼ 24	237 (29.22%)
25 ∼ 26	220 (27.13%)
27 ∼ 28	216 (26.63%)
Surgical method^2^	
BCS	388 (47.84%)
MRM	423 (52.16%)
CSI^3^	
Yes	195 (24.04%)
No	616 (75.96%)
Chemotherapy regimens^4^	
AC-T	383 (47.23%)
TCb	151 (18.62%)
TAC	111 (13.69%)
TC	101 (12.45%)
AC	65 (8.01%)

^1^ Tumor staging was performed in accordance with the 8th edition of the American Joint Committee on Cancer (AJCC) staging system (17). ^2^BCS, breast-conserving surgery; MRM: modified radical mastectomy. ^3^CSI, chest wall + supraclavicular + internal mammary lymph node irradiation. ^4^AC-T, anthracycline plus cyclophosphamide followed by taxane; TCb, taxane plus carboplatin; TAC, taxane plus anthracycline plus cyclophosphamide; TC, taxane plus cyclophosphamide; AC, anthracycline plus cyclophosphamide.

### 2.2 CT scanning

Patients were immobilized in the supine position using a head-neck-thorax thermoplastic mold. CT scans were performed on the Somatom Confidence CT scanner (Siemens, Germany), with the scanning range extending from the mandible to the inferior border of the liver and a slice thickness of 3 mm.

### 2.3 Contouring

Gross target volume (GTV) and organ at risk (OAR) were defined according to International Commission on Radiation Units & Measurement Report 50 and 83 (18, 19). The GTV was expanded by a margin based on the breast cancer setup errors previously documented in our department (20), and designated as the PTV. Namely, GTV was expanded by 5 mm isotropically to form CTV, which was further expanded by 5 mm isotropically to generate PTV. OARs, including both lungs, heart, contralateral breast, ipsilateral humeral head, spinal cord and thyroid gland, were delineated.

### 2.4 Treatment planning, verification and delivery

Plans were created for all patients using the Eclipse treatment planning system (Varian Medical Systems, Version 15.6, USA). The dose objectives for PTV and dose constraints for OARs were summarized in **Table 2**.

**Table 2:** The dose objectives for PTV and dose constraints for OARs.

Structures		Parameters	Dose objectives
PTV^1^			
	BCS	D_95%_	≥50 Gy
	MRM	D_95%_	≥50 Gy
PTV_tb_^2^			
	BCS	D_95%_	≥10 Gy
Ipsilateral lung			
		V_20 Gy_	≤25% ∼ 28%
		V_5 Gy_	≤50% ∼ 60%
		MLD^3^	≤15 Gy
Heart			
		D_mean_	≤4 Gy ∼ 8 Gy
		V_5 Gy_	≤30% ∼ 40%
Contralateral lung			
		V_5 Gy_	≤20%
Contralateral breast			
		V_5 Gy_	≤20%
Thyroid gland			
		D_mean_	≤20 Gy
Spinal cord			
		D_max_	≤20 Gy ∼ 30 Gy

^1^ BCS, breast-conserving surgery; MRM, modified radical mastectomy; ^2^tb, tumor bed; ^3^MLD, mean lung dose.

For patients underwent MRM, the prescribed dose was 50 Gy in 25 fractions to the PTV. Patients after BCS received 50 Gy in 25 fractions to the PTV followed by a sequential boost of 10 Gy in 5 fractions to the tumor bed. All plans were reviewed and approved by senior medical physicists and radiation oncologists. Furthermore, prior to the first fraction of radiotherapy, all treatment plans underwent gamma passing rate verification using the SNC Patient gamma analysis software (SUN NUCLEAR Corporation) on the ArcCheck 3-dimensional verification system, performed by qualified medical physicists. With the gamma passing rate evaluated under 3 mm/3% criteria, the passing rates ranged from 98.4% to 100%; under 2 mm/2% criteria, the rates ranged from 96.0% to 100%, all results meeting the recommendations of American Association of Physicists in Medicine (AAPM) Task Group 218 (TG-218) Report (21). All plans were deemed clinically acceptable for treatment. All patients received radiotherapy on a Varian Halcyon v2.0 linear accelerator, and cone-beam computed tomography (CBCT) image guidance was performed before each fraction.

### 2.5 Radiation pneumonitis diagnosis, grading, follow-up, image interpretation and differential diagnosis

RP was diagnosed and graded in strict adherence to the CTCAE V5.0. All enrolled patients followed a standardized follow-up regimen uniformly. Clinical symptom assessment was performed at one month (30 days ∼ 32 days) after the completion of radiotherapy, and routine chest computed tomography (CT) surveillance was scheduled at the 3^rd^ month (90 days ∼ 92 days) and 6^th^ month (180 days ∼ 182 days) post-treatment; no additional imaging examinations were conducted for any participant.

All chest CT images were independently interpreted and graded by one senior radiologist and one senior radiation oncologist under double-blinded conditions. In the event of interpretive discrepancies, a third senior specialist was consulted for joint review to reach a unanimous conclusion.

Rigorous differential diagnostic procedures were implemented to distinguish RP from other pulmonary disorders. Pulmonary infection was ruled out based on the presence of fever and elevated inflammatory biomarkers. Pulmonary lymphangitis and other interstitial lung abnormalities were differentiated according to imaging manifestations, medical history and clinical course. A definitive diagnosis of RP was established only when patients fully met the diagnostic criteria of CTCAE V5.0.

### 2.6 Statistical methods

Data analysis was performed using IBM SPSS Statistics 27 software. For univariate analysis, the t-test or the Mann-Whitney U test was used for continuous variables, while the chi-square test was applied to categorical data. Multivariate analysis was performed using binary logistic regression to identify independent risk factors for RP. Receiver operating characteristic (ROC) curve analysis was conducted to evaluate the predictive utility of these factors, with the area under the curve (AUC) quantified to assess diagnostic accuracy. A *P*-value < 0.05 was considered statistically significant.

## 3 Results

According to the CTCAE 5.0 criteria for RP, among 811 enrolled patients, 36 (4.44%) exhibited grade 1 RP, 13 (1.60%) had grade 2 RP, and no grade 3 or higher toxicity was documented. The cumulative incidence of RP at 1 month, 3 months, and 6 months after radiotherapy was 0.99% (8/811), 4.69% (38/811), and 6.04% (49/811), respectively. The majority of RP cases (38/49) occurred within 3 months after radiotherapy.

Univariate analysis of categorical variables (surgical approach, tumor stage, chemotherapy regimens, chemotherapy-radiotherapy interval time, CSI) demonstrated that RP in breast cancer patients was not significantly associated with surgical approach (*P* = 0.057), chemotherapy regimens (*P* = 0.350) and chemotherapy-radiotherapy interval time (*P* = 0.533), but was significantly correlated with tumor stage (*P* = 0.010) and CSI (*P* < 0.001), as detailed in **Table 3**.

**Table 3:** Univariate analysis of categorical variables in 811 patients with radiation pneumonitis.

Variables		RP (n)	nRP^1^ (n)	χ^2^	*P*
Surgical approach^2^				3.613	0.057
	BCS	17	371		
	MRM	32	391		
Tumor stage				9.213	0.010
	I	16	196		
	II	11	337		
	III	22	229		
Chemotherapy regimens^3^				4.437	0.350
	AC-T	28	355		
	TCb	11	140		
	TAC	4	107		
	TC	4	97		
	AC	2	63		
Chemotherapy-radiotherapy interval time				2.196	0.533
	21 ∼ 22 days	12	126		
	23 ∼ 24 days	14	223		
	25 ∼ 26 days	12	208		
	27 ∼ 28 days	11	205		
CSI^4^				115.908	<0.001
	Yes	43	152		
	No	6	610		

^1^ nRP, non-RP.

^2^ BCS. breast-conserving surgery; MRM, modified radical mastectomy.

^3^ AC-T, anthracycline plus cyclophosphamide followed by taxane; TCb, taxane plus carboplatin; TAC, taxane plus anthracycline plus cyclophosphamide; TC, taxane plus cyclophosphamide; AC, anthracycline plus cyclophosphamide

^4^ CSI, chest wall + supraclavicular + internal mammary lymph node irradiation.

Univariate analysis of continuous variables demonstrated that RP was not significantly associated with age and the volume of PTV (all *P* > 0.05). Conversely, the number of chemotherapy cycle, the ipsilateral lung V_5_, V_10_, V_20_ and MLD were all significantly correlated with RP (all *P* < 0.05). Details are presented in **Table 4**.

**Table 4:** Univariate analysis of continuous variables in 811 patients with radiation pneumonitis.

Variables		RP	nRP^1^	t/U^*^	*P*
Age		52.88 ± 9.07	52.58 ± 9.56	0.183	0.855
PTV volume		568.97 ± 236.24	540.82 ± 209.54	0.766	0.444
Chemotherapy cycles^*^		8 (7∼8)	6 (6∼7)	7072.500	<0.001
Ipsilateral lung	MLD^2^(Gy)	13.40 ± 0.80	10.41 ± 1.56	22.208	<0.001
	V_5_(%)	55.21 ± 3.29	45.80 ± 7.08	16.878	<0.001
	V_10_(%)	37.10 ± 2.56	29.20 ± 5.32	17.978	<0.001
	V_20_(%)	23.84 ± 2.12	17.44 ± 3.64	17.809	<0.001

^1^nRP, non-RP; ^2^MLD, mean lung dose; ^*^U, The Mann-Whitney U test.

Considering that ipsilateral lung V_5_, V_10_, V_20_ and MLD are inherently highly correlated. We performed collinearity diagnosis via Variance Inflation Factor (VIF) before multivariate modeling. The results showed that VIF values of V_10_ and V_20_ were greater than 10, indicating severe collinearity. In order to avoid distortion of model regression caused by severe collinearity, we first excluded the dosimetric parameters with VIF > 10 (V_10_, V_20_), and then retained the MLD with the lowest VIF (4.819) among the remaining indicators. The results of multicollinearity diagnostics for dosimetric parameters are summarized in **Table 5**.

**Table 5:** The results of multicollinearity diagnostics for dosimetric parameters.

Parameters	Collinearity statistics	
	Tolerance	VIF^2^
Constant		
V_5_	0.166	6.017
V_10_	0.067	14.858
V_15_	0.054	18.586
MLD^1^	0.208	4.819

^1^MLD, mean lung dose; ^2^VIF, Variance Inflation Factor.

Finally, MLD, tumor stage, CSI and the number of chemotherapy cycles were incorporated into the subsequent multivariate analysis. Given the evidence that CSI is a causal factor for increased MLD, and the two variables share the same causal pathway, we established separate models and discuss mediating effects. The detailed results are summarized in **Table 6**.

**Table 6:** Mediation effect analysis of CSI and MLD on radiation pneumonitis.

Effect type	Total effect	Direct effect	Indirect effect (CSI^1^→MLD^2^→RP^3^)
Coefficient B	3.116	1.732	3.768
Exp(B)	22.560	5.654	43.305
95% BCa CI^4^	9.480∼53.694	1.768∼18.080	--
*P*	<0.001	0.003	<0.001
Proportion of mediation effect	--	--	120.924%

The 95% confidence interval of the indirect effect was calculated using 1000 bias-corrected and accelerated (BCa) bootstrap resamples. ^1^CSI, chest wall + supraclavicular + internal mammary lymph node irradiation; ^2^MLD: mean lung dose; ^3^RP, radiation pneumonitis; ^4^BCa CI, bias-corrected and accelerated confidence interval.

It can be seen from **Table 6** that multivariate logistic regression analysis without adjusting for MLD revealed that CSI was associated with a 22.560-fold increased risk of RP. After adjusting for MLD, the direct effect of CSI on RP remained significant but was greatly attenuated (OR = 5.654, 95%CI: 1.768∼18.080, *P* = 0.003).

Additional multivariate analysis including both CSI and MLD are presented in **Table 7**. The results confirmed that MLD was the strongest predictor of RP (OR = 11.136, 95%CI: 5.494∼22.571, *P* < 0.001), while CSI and chemotherapy cycles remained independent predictors.

**Table 7:** Results of the multivariate analysis for radiation pneumonitis.

Variables	Chemotherapy cycles	MLD^1^	CSI^2^	Tumor stage
RCB^3^	1.008	2.410	1.732	0.061
Standard error	0.275	0.360	0.593	0.325
Wald value	13.415	44.710	8.531	0.036
*P*	<0.001	<0.001	0.003	0.850
Exp(B)	2.739	11.136	5.654	1.063
95%CI^3^	1.597∼4.696	5.494∼22.571	1.768∼18.080	0.562∼2.011

^1^ MLD, mean lung dose; ^2^CSI, chest wall + supraclavicular + internal mammary lymph node irradiation; ^3^RCB, Regression coefficient B; ^4^CI, Confidence Interval.

**Figure 2:**
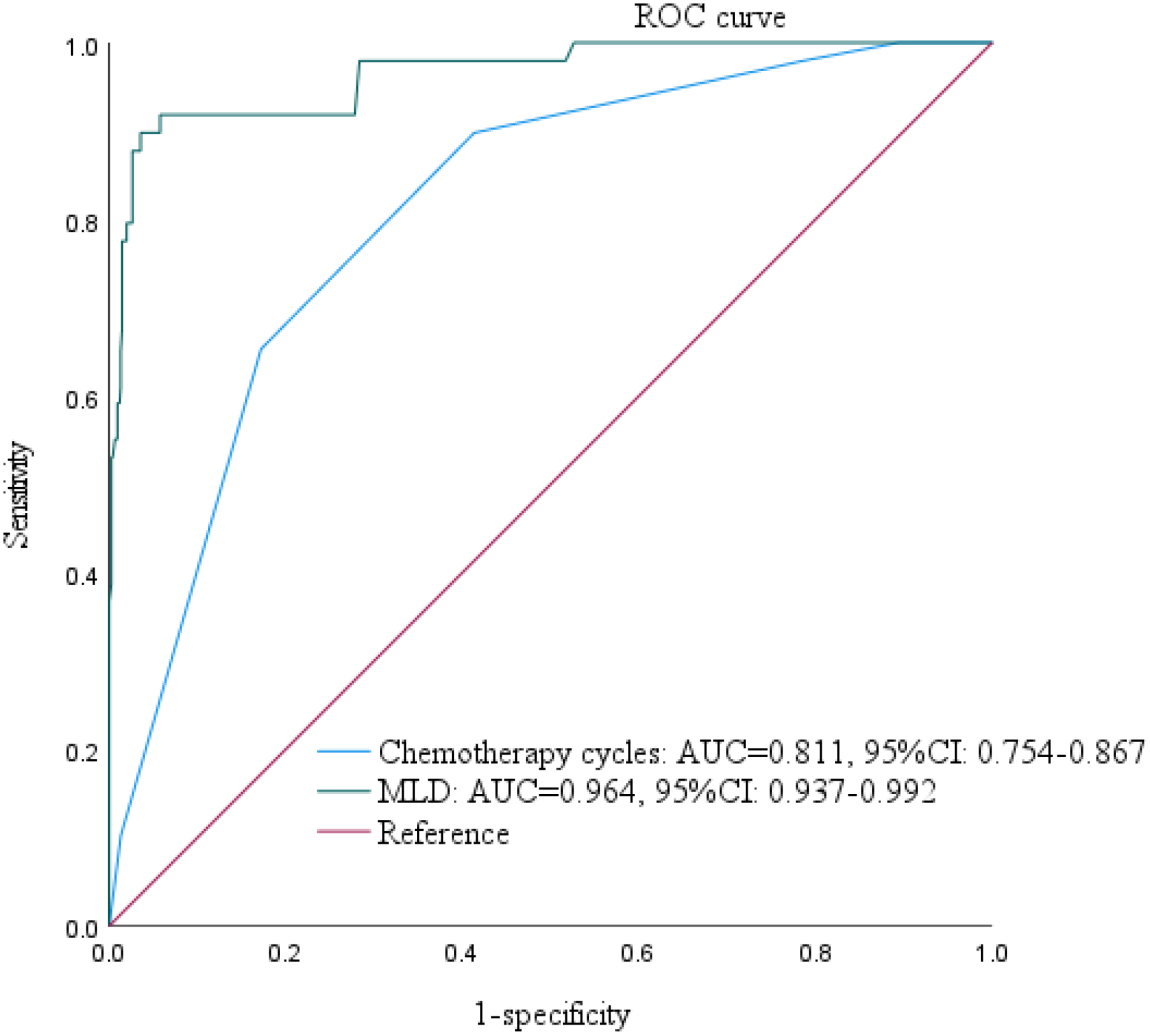
Receiver operating characteristic curves for chemotherapy cycles and MLD.

ROC analysis was conducted to evaluate MLD and the number of chemotherapy cycles. Results demonstrated that for the number of chemotherapy cycles, the area under the curve (AUC) for RP was 0.811, with a 95% confidence interval (95% CI) of 0.754 to 0.867. When a cutoff value of 7 was applied to the number of chemotherapy cycles, the predictive sensitivity and specificity for RP were 0.898 and 0.587, respectively. Specifically, 196 patients (24.17%) received 7 cycles, 148 patients (18.25%) received 8 cycles, and 15 patients (1.85%) received 9 cycles. For MLD, the AUC for RP was 0.964, with a 95% CI of 0.937 to 0.992. Using a cutoff value of 12.70 Gy to MLD, the predictive sensitivity and specificity for RP were 0.898 and 0.965, respectively. The ROC curves are presented in **Figure 2**.

To evaluate the robustness and generalizability of the multivariate prediction model, we performed internal validation using the bootstrap method with 1000 resamples. As shown in **Table 8**, the bootstrap-corrected AUC of MLD and chemotherapy cycles were close to the original AUC with a Brier score were 0.025 for MLD and 0.052 for cycles.

**Table 8:** Bootstrap bias-corrected internal validation results of the MLD and cycles.

Parameters	MLD	Chemotherapy cycles
Validation method	Bootstrap 1000 resampling	Bootstrap 1000 resampling
Apparent AUC	0.964	0.811
Corrected AUC	0.965	0.810
95%CI	0.926∼0.995	0.737∼0.881
Brier score	0.025	0.052
Hosmer-Lemeshow χ^2^	31.253	1.109
Hosmer-Lemeshow df	8	3
Hosmer-Lemeshow *P*	<0.001	0.775

**Figure 3:**
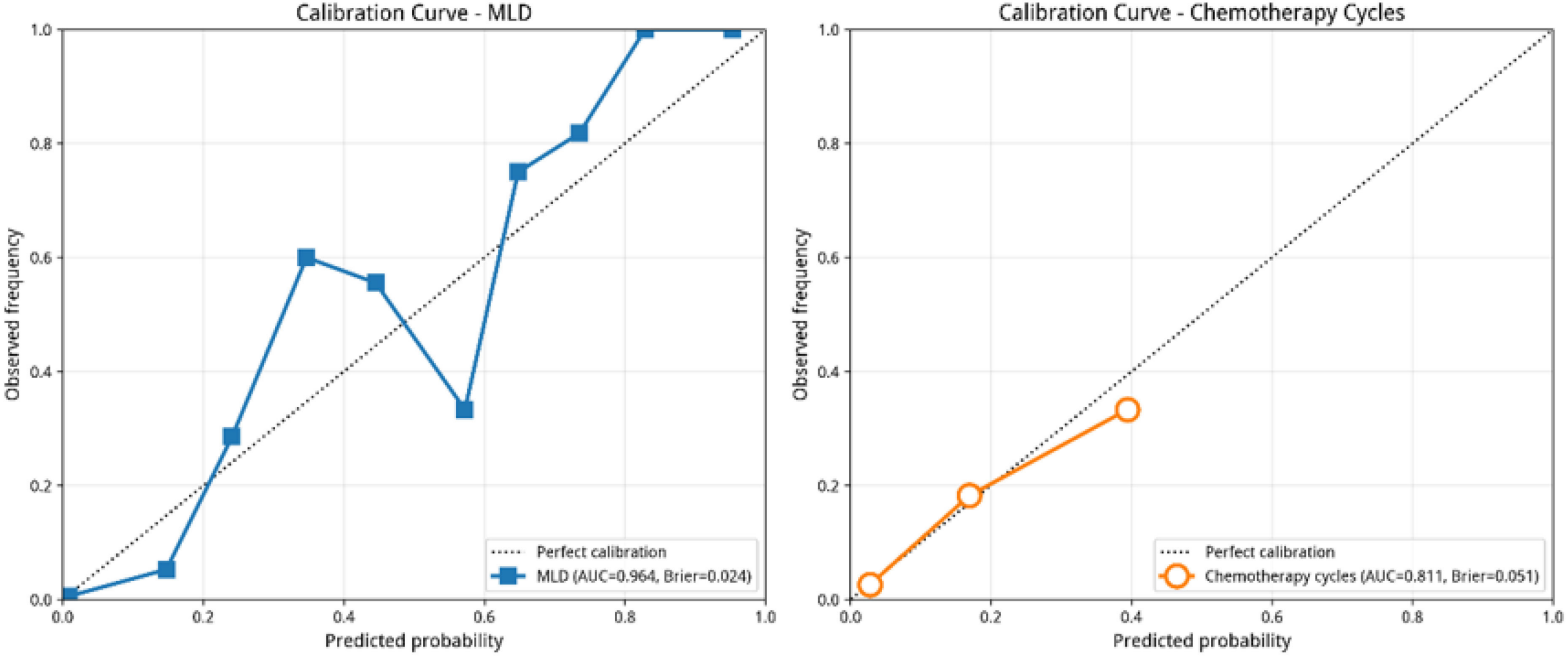
Calibration curves of the prediction models.

The calibration curves of the prediction models are in **Figure 3**.

For CSI, since CSI is a binary variable and thus has only one valid cut-off point, we used a contingency table to analyze the association of CSI with the outcome. As shown in **Table 9**, patients receiving CSI had a significantly higher risk of RP compared to those not receiving CSI (OR = 28.761, 95%CI: 12.020∼68.817, *P* < 0.001). Additionally, the sensitivity and specificity were 0.878 and 0.810, respectively. And the AUC was 0.839.

**Table 9:** Contingency tables for CSI.

	RP=0^a^	RP=1^b^	Total	Ratio
CSI=0 ^a^	6	610	616	0.974%
CSI=1 ^b^	43	152	195	22.051%

^a^ No; ^b^Yes.

## 4 Discussions

RP remains a significant dose-limiting toxicity in thoracic radiotherapy, with incidence and severity modulated by both treatment-related and patient-specific factors. At present, in accordance with clinical guidelines and related research for the diagnosis and management of radiation-associated pneumonitis, definitive diagnosis necessitates immediate suspension of radiotherapy and initiation of targeted therapeutic interventions (22, 23). The overall therapeutic principle involves administering glucocorticoid therapy with sufficient dosage, adequate course, and individualized regimen. The use of glucocorticoids should follow a gradual tapering principle to prevent rebound of RP. Additionally, in the event of pulmonary infection, empirical antimicrobial therapy with antibiotics should be initiated promptly. In addition to glucocorticoid and antibiotic therapies, adjunctive treatments such as antitussive and expectorant medications, oxygen therapy, or nebulization should be administered based on the patient’s clinical symptoms.

Consistent with previous reports (24–26), our findings underscore the critical role of lung dosimetry, demonstrating that MLD is the most robust independent predictor of RP in breast cancer radiotherapy (AUC=0.964). This reinforces the clinical imperative to rigorously minimize MLD during plan optimization. In the study, the cut-off value of MLD was 12.70 Gy, which was lower than the 13 ∼ 20 Gy dose constraint proposed in the QUANTEC guidelines (27). The primary reason for this discrepancy is that the QUANTEC guideline was derived from three-dimensional conformal radiotherapy (3D-CRT) and designed to limit the risk of grade ≥2 symptomatic RP to below 10% ∼ 20%. In contrast, this study was based on intensity-modulated radiotherapy (IMRT), and all RP cases observed in the cohort were grade 1 or 2.

The association between CSI and elevated RP risk (OR = 5.654) is particularly salient, as irradiation of the internal mammary chain inevitably increases the volume of ipsilateral lung receiving low- to moderate-dose exposure, thereby augmenting the effective lung dose. The mediation analysis demonstrated that the majority of the effect of CSI on RP was mediated through elevated MLD, which is consistent with previous studies. Therefore, dose optimization strategies aimed at reducing MLD should be the primary approach to decrease the risk of RP in patients receiving CSI irradiation. The independent predictive value of chemotherapy cycles further highlights the synergistic toxicity of cytotoxic agents and radiation, potentially through subclinical lung injury that sensitizes parenchyma to radiation-induced inflammation. The identified threshold of >7 cycles provides a practical clinical benchmark for heightened vigilance and more stringent dosimetric constraints in this subgroup.

Mitigation of RP risk may be achieved through advanced radiotherapy techniques. Respiratory motion management, such as deep inspiration breath-hold (DIBH), has been demonstrated to significantly reduce cardiac and pulmonary doses, thereby lowering RP probability (28, 29). Furthermore, the dosimetric advantages inherent to proton therapy, characterized by a steep Bragg peak and absence of exit dose, confer substantial normal tissue sparing. Comparative studies of proton versus photon plans have shown marked reductions in lung V_20_ and MLD, even in complex clinical scenarios such as bilateral breast cancer or comprehensive nodal irradiation (30, 31). These modalities represent promising strategies for further reducing RP incidence in high-risk populations identified by the present study.

Nevertheless, this study has several limitations. First, 73.5% of RP cases were asymptomatic CTCAE Grade 1 subclinical lesions. Although we adopted standardized follow-up, double-blind image review and strict differential diagnosis to reduce surveillance and ascertainment bias, subjective interpretation of imaging findings may still exist. Secondly, the number of clinically meaningful symptomatic RP (Grade ≥ 2) was only 13 cases, with insufficient sample size for grade-stratified analysis. Therefore, we combined all RP grades for statistical analysis in this study. And then, the incidence of RP in patients without CSI was extremely low, resulting in sparse cells in the contingency table and a relatively wide 95% confidence interval of the OR value for CSI. Fourth, this study did not explore the potential interaction effects among the three identified risk factors due to the relatively small sample size of the high-risk subgroup with all three characteristics. Finally, our study excluded patients with a history of smoking, pre-existing cardiopulmonary diseases, and autoimmune disorders. It also limits the external validity of our results.

In addition, it is more noteworthy that the exceptionally high discriminative performance of MLD reported in this study must be interpreted strictly within the specific context of its generation. First, the rigorous institutional planning dose constraints may have inadvertently enriched a patient subset with a highly prominent threshold effect, thereby leading to the exaggerated manifestation of the model’s predictive performance in this cohort. Second, the inherent limitations of a single-center retrospective design, coupled with potential unmeasured confounding factors, preclude the direct generalization of this threshold and the associated AUC value to other clinical centers with divergent treatment protocols and greater population heterogeneity. Finally, from a biological standpoint, RP is a complex multifactorial pathological process. The near-perfect predictive accuracy achieved by a single dosimetric parameter further indicates that the observed association may be amplified by the specific case composition of our study sample. Therefore, we conservatively assert that while MLD represents an extremely potent risk stratification tool, its utility as a standalone predictive model urgently requires rigorous external validation in prospective cohorts employing different dose constraint strategies and encompassing more diverse patient populations.

In the next phase, the study will continue to enroll more breast cancer patients receiving radiotherapy to continue to investigate the problem of RP and to address the study limitations mentioned earlier. More importantly, we also plan to incorporate multiple factors closely associated with lung toxicity and treatment intensity, including molecular subtype, HER2 status, anti-HER2 targeted therapy, endocrine therapy, DIBH, radiotherapy techniques and tumor laterality to improve the generalizability of the model.

## 5 Conclusions

RP is one of the most prevalent complications in thoracic malignancy radiotherapy, imposing constraints on radiation dosage and compromising treatment efficacy while deteriorating patient quality of life. Notably, RP manifests with low incidence (typically <5%) and mild symptomatology in breast cancer radiotherapy, thus the relevant research into breast cancer-specific RP remain scarce. In this study, 49 out of 811 patients developed RP (grade 1: 36, grade 2: 13), representing a 6.04% incidence rate. Multivariate analysis revealed that the number of chemotherapy cycles, MLD and CSI were risk factors for RP. It is therefore recommended that clinicians strictly constrain the MLD, particularly in patients with a history of more than 7 chemotherapy cycles who are also receiving chest wall + supraclavicular + internal mammary lymph node irradiation.

## Data Availability

The original contributions presented in the study are included in the article/supplementary material. Further inquiries can be directed to the corresponding author.

## References

[1] Bray F Laversanne M Sung H Ferlay J Siegel RL Soerjomataram I . Global cancer statistics 2022: GLOBOCAN estimates of incidence and mortality worldwide for 36 cancers in 185 countries. CA Cancer J Clin. (2024) 74:229–63. doi: 10.3322/caac.21834 38572751

[2] Giaquinto AN Sung H Miller KD Kramer JL Newman LA Minihan A . Breast cancer statistics, 2022. CA: A Cancer J For Clin. (2022) 72:524–41. doi: 10.3322/caac.21754 36190501

[3] Chen L Li H Zhang H Yang H Qian J Li Z . Camrelizumab vs placebo in combination with chemotherapy as neoadjuvant treatment in patients with early or locally advanced triple-negative breast cancer: The CamRelief randomized clinical trial. JAMA. (2025) 333:673–81. doi: 10.1001/jama.2024.23560 39671272 PMC11862970

[4] Lowry KP Geuzinge HA Stout NK Alagoz O Hampton J Kerlikowske K . Breast cancer screening strategies for women with ATM, CHEK2, and PALB2 pathogenic variants: A comparative modeling analysis. JAMA Oncol. (2022) 8:587–96. doi: 10.1001/jamaoncol.2021.6204 35175286 PMC8855312

[5] Piruzan E Vosoughi N Mahdavi SR Khalafi L Mahani H . Target motion management in breast cancer radiation therapy. Radiol Oncol. (2021) 55:393–408. doi: 10.2478/raon-2021-0040 34626533 PMC8647788

[6] Yin H Jia W Yu J Zhu H . Radiation pneumonitis after concurrent aumolertinib and thoracic radiotherapy in EGFR-mutant non-small cell lung cancer patients. BMC Cancer. (2024) 24:197. doi: 10.1186/s12885-024-11946-y 38347438 PMC10863168

[7] Hanania AN Mainwaring W Ghebre YT Hanania NA Ludwig M . Radiation-induced lung injury: Assessment and management. Chest. (2019) 156:150–62. doi: 10.1016/j.chest.2019.03.033 30998908 PMC8097634

[8] Gueiderikh A Sarrade T Kirova Y De La Lande B De Vathaire F Auzac G . Radiation-induced lung injury after breast cancer treatment: Incidence in the CANTO-RT cohort and associated clinical and dosimetric risk factors. Front Oncol. (2023) 13:1199043. doi: 10.3389/fonc.2023.1199043 37456251 PMC10342531

[9] Freites-Martinez A Santana N Arias-Santiago S Viera A . Using the Common Terminology Criteria for Adverse Events (CTCAE - Version 5.0) to evaluate the severity of adverse events of anticancer therapies. Actas Dermosifiliogr (Engl Ed). (2021) 112:90–2. doi: 10.1016/j.ad.2019.05.009 32891586

[10] Bölke E Matuschek C . Images in clinical medicine. Radiation pneumonitis after radiotherapy for breast cancer. N Engl J Med. (2009) 361:e65. doi: 10.1056/NEJMicm0810650 20042752

[11] Yu TK Whitman GJ Thames HD Buzdar AU Strom EA Perkins GH . Clinically relevant pneumonitis after sequential paclitaxel-based chemotherapy and radiotherapy in breast cancer patients. J Natl Cancer Inst. (2004) 96:1676–81. doi: 10.1093/jnci/djh315 15547180

[12] Jagsi R Griffith KA Boike TP Walker E Nurushev T Grills IS . Differences in the acute toxic effects of breast radiotherapy by fractionation schedule: Comparative analysis of physician-assessed and patient-reported outcomes in a large multicenter cohort. JAMA Oncol. (2015) 1:918–30. doi: 10.1001/jamaoncol.2015.2590 26247417

[13] Coles CE Haviland JS Kirby AM Griffin CL Sydenham MA Titley JC . Dose-escalated simultaneous integrated boost radiotherapy in early breast cancer (IMPORT HIGH): A multicentre, phase 3, non-inferiority, open-label, randomised controlled trial. Lancet. (2023) 401:2124–37. doi: 10.1016/S0140-6736(23)00619-0 37302395

[14] Bardia A Hu X Dent R Yonemori K Barrios CH O'Shaughnessy JA . Trastuzumab deruxtecan after endocrine therapy in metastatic breast cancer. N Engl J Med. (2024) 391:2110–22. doi: 10.1056/NEJMoa2407086 39282896

[15] Park JB Jang BS Chang JH Kim JH Choi CH Hong KY . The impact of the new ESTRO-ACROP target volume delineation guidelines for postmastectomy radiotherapy after implant-based breast reconstruction on breast complications. Front Oncol. (2024) 14:1373434. doi: 10.3389/fonc.2024.1373434 38846971 PMC11153655

[16] Rachel A . Efficacy, toxicity, and cosmesis of partial breast irradiation: Honing in on dose and patient selection. JCO. (2025) 43:481–3. doi: 10.1200/JCO-24-01625 39541559

[17] Giuliano AE Connolly JL Edge SB Mittendorf EA Rugo HS Solin LJ . Breast cancer-major changes in the American Joint Committee on Cancer eighth edition cancer staging manual. CA Cancer J Clin. (2017) 67:290–303. doi: 10.3322/caac.21393 28294295

[18] Hodapp N . Der ICRU-Report 83: Verordnung, Dokumentation und Kommunikation der fluenzmodulierten Photonenstrahlentherapie (IMRT). Strahlenther Onkol. (2012) 188:97–100. doi: 10.1007/s00066-011-0015-x 22234506

[19] Grégoire V Mackie T . State of the art on dose prescription, reporting and recording in intensity-modulated radiation therapy (ICRU report No. 83). Cancer / Radiothérapie. (2011) 15:555–9. doi: 10.1016/j.canrad.2011.04.003 21802333

[20] Yin C Deng J Mei G Cheng H He Y Liu J . Comparison of plan quality and robustness using VMAT and IMRT for breast cancer. Open Phys. (2024) 22:20240026. doi: 10.1515/phys-2024-0026 31755547

[21] Miften M Olch A Mihailidis D Moran J Pawlicki T Molineu A . Tolerance limits and methodologies for IMRT measurement-based verification QA: Recommendations of AAPM Task Group No. 218. Med Phys. (2018) 45:e53–83. doi: 10.1002/mp.12810 29443390

[22] Jain V Berman AT . Radiation pneumonitis: Old problem, new tricks. Cancers (Basel). (2018) 10:222. doi: 10.3390/cancers10070222 29970850 PMC6071030

[23] Ullah T Patel H Pena GM Shah R Fein AM . A contemporary review of radiation pneumonitis. Curr Opin Pulm Med. (2020) 26:321–5. doi: 10.1097/MCP.0000000000000682 32427626

[24] Karlsen J Tandstad T Sowa P Salvesen Stenehjem JS Lundgren S . Pneumonitis and fibrosis after breast cancer radiotherapy: Occurrence and treatment-related predictors. Acta Oncol. (2021) 60:1651–8. doi: 10.1080/0284186X.2021.1976828 34618657

[25] Lee BM Chang JS Kim SY Keum KC Suh CO Kim YB . Hypofractionated radiotherapy dose scheme and application of new techniques are associated to a lower incidence of radiation pneumonitis in breast cancer patients. Front Oncol. (2020) 10:124. doi: 10.3389/fonc.2020.00124 32117771 PMC7026386

[26] Blom Goldman U Anderson M Wennberg B Lind P . Radiation pneumonitis and pulmonary function with lung dose-volume constraints in breast cancer irradiation. J Radiother Pract. (2014) 13:211–7. doi: 10.1017/S1460396913000228 24910536 PMC4045177

[27] Bentzen SM Constine LS Deasy JO Eisbruch A Jackson A Marks LB . Quantitative analyses of normal tissue effects in the clinic (QUANTEC): An introduction to the scientific issues. Int J Radiat Oncol Biol Phys. (2010) 76:S3–9. doi: 10.1016/j.ijrobp.2009.09.040 20171515 PMC3431964

[28] Jingu K Ito K Sato K Umezawa R Yamamoto T Takahashi N . VMAT with DIBH in hypofractionated radiotherapy for left-sided breast cancer after breast-conserving surgery: Results of a non-inferiority clinical study. J Radiat Res. (2024) 65:87–91. doi: 10.1093/jrr/rrad096 38091980 PMC10803169

[29] Peters GW Gao SJ Knowlton C Zhang A Evans SB Higgins S . Benefit of deep inspiratory breath hold for right breast cancer when regional lymph nodes are irradiated. Pract Radiat Oncol. (2022) 12:e7–e12. doi: 10.1016/j.prro.2021.08.010 34508890

[30] Brooks ED Mailhot Vega RB Vivers E Burchianti T Liang X Spiguel LR . Proton therapy for bilateral breast cancer maximizes normal-tissue sparing. Int J Part Ther. (2023) 9:290–301. doi: 10.14338/IJPT-22-00041.1 37169011 PMC10166012

[31] Fattahi S Mullikin TC Aziz KA Afzal A Smith NL Francis LN . Proton therapy for the treatment of inflammatory breast cancer. Radiother Oncol. (2022) 171:77–83. doi: 10.1016/j.radonc.2022.04.008 35436537

